# Genetic Predisposition and Inflammatory Inhibitors in COVID-19: Where Do We Stand?

**DOI:** 10.3390/biomedicines10020242

**Published:** 2022-01-24

**Authors:** Marios Sagris, Panagiotis Theofilis, Alexios S. Antonopoulos, Evangelos Oikonomou, Kostas Tsioufis, Dimitris Tousoulis

**Affiliations:** 1 1st Cardiology Clinic, School of Medicine, “Hippokration” General Hospital, National and Kapodistrian University of Athens, 157 72 Athens, Greece; panos.theofilis@hotmail.com (P.T.); alexios.antonopoulos@cardiov.ox.ac.uk (A.S.A.); boikono@gmail.com (E.O.); ktsioufis@gmail.com (K.T.); drtousoulis@hotmail.com (D.T.); 2 3rd Department of Cardiology, “Sotiria” Thoracic Diseases Hospital of Athens, University of Athens Medical School, 157 72 Athens, Greece

**Keywords:** COVID-19, SARS-CoV-2, inflammation, genetics, cytokines

## Abstract

Severe acute respiratory syndrome Coronavirus-2 (SARS-CoV-2) and the resulting coronavirus disease-19 (COVID-19) have led to a global pandemic associated with high fatality rates. COVID-19 primarily manifests in the respiratory system as an acute respiratory distress syndrome following viral entry through the angiotensin-converting enzyme-2 (ACE2) that is present in pulmonary epithelial cells. Central in COVID-19 is the burst of cytokines, known as a “cytokine storm”, and the subsequent widespread endothelial activation, leading to cardiovascular complications such as myocarditis, arrhythmias, and adverse vascular events, among others. Genetic alterations may play an additive, detrimental role in the clinical course of patients with COVID-19, since gene alterations concerning ACE2, major histocompatibility complex class I, and toll-like receptors may predispose patients to a worse clinical outcome. Since the role of inflammation is quintessential in COVID-19, pharmacologic inhibition of various signaling pathways such as the interleukin-1 and -6, tumor necrosis factor-alpha, interferon gamma, Janus kinase-signal transducer and activator of transcription, and granulocyte–macrophage colony-stimulating factor may ameliorate the prognosis following timely administration. Finally, frequently used, non-specific anti-inflammatory agents such as corticosteroids, statins, colchicine, and macrolides represent additional therapeutic considerations.

## 1. Introduction

Coronaviruses (CoVs) are RNA viruses with a single strand that belong to the Coronaviridae family while four CoVs categories have thus far been identified: α, β, γ, δ. SARS-CoV2 penetrates human cells by attaching to the angiotensin-converting enzyme 2 (ACE2), abundant in alveolar lung cells, vascular endothelium, cardiac myocytes, and other cells [1]. The novel coronavirus disease 2019 (COVID-19) emerged as a severe acute respiratory illness and was proclaimed a pandemic on 30 January 2020, affecting primarily the residents of Wuhan, Hubei Province in China [2,3].

The course of the disease is mild in the large proportion of patients, while severe cases with hospitalization and high mortality rates also occur. Physicians have tried to classify the disease course by dividing it into four stages [4,5]. In the first stage (Stage I), fever, dry cough, tiredness, and myalgia are the most common symptoms which are, however, not specific to the disease. In the second stage (Stage II), bilateral pulmonary parenchymal ground-glass and consolidative pulmonary opacities are presented on the computed tomography scan in the vast majority of COVID-19 patients with viral pneumonia [4]. A hypercoagulable state has been observed, especially in hospitalized patients in Stage III of the disease, driven by abnormal coagulation cascades’ activation [5]. Finally, multiorgan failure on top of excessive hypoxemia appears in Stage IV with hyperresponsiveness of the immune system [4,5]. This stage is characterized by rapid elevation of inflammatory circulating cytokines such as interleukin (IL)-1, IL-2, IL-6, and IL-7, tumor necrosis factor (TNF)-α, granulocyte–macrophage colony-stimulating factor (GM–CSF), macrophage inflammatory protein 1-α (MIP-1α), C-reactive protein (CRP), ferritin, and D-dimer [6]. This extreme inflammatory response causes severe adult Acute Respiratory Distress Syndrome (ARDS) and the so-called “cytokine storm” [6].

As such, the mitigation of the excessive inflammatory immune response is of high scientific interest and clinical relevance. Recently, anti-inflammatory drugs and immunomodulators have been studied as potential therapies to minimize cardiovascular and systemic adverse effects.

## 2. Inflammatory Mechanisms in COVID-19-Related Cardiovascular Disorders

A significant relationship has been established between SARS-CoV-2 and the Renin–Angiotensin System (RAS), which governs renal, cardiovascular, and immunological functions [7]. This relationship is reinforced by the ability of SARS-CoV-2 to bind to the metallopeptidase ACE2 via its spike glycoprotein S [7]. ACE2 is found on the surface of vascular epithelial cells, lung, kidney, gut, heart, and brain neurons and immune monocytes/macrophages [7]. COVID-19 is considered a lung-centric viral infection with direct adverse effects on vessels and the heart via various putative pathways [8]. To begin with, hypoxemia produced by lung injury results in a gradual decrease in circulating oxygen partial pressure and saturation. Oxygen free radicals, lactic acid, and metabolite aggregation develop due to cardiac cell damage [8]. Clinical findings have demonstrated that SARS-CoV-2 binds to ACE2, causing an increase in Angiotensin II release via the RAS, resulting in excessive vasoconstriction. This stresses the myocardial and vascular systems by increasing cardiac loading, which leads to left ventricular hypertrophy and elevated blood pressure over time [1,9]. Myocardial autopsies of COVID-19 patients who suffered heart attacks highlighted the probability of viral myocarditis. SARS-CoV-2 myocarditis has been reported as a significant acute ventricular dysfunction coupled with widespread myocardial edema, while cases of fulminant evolution, complicated with pericarditis, pericardial effusion, and consequent cardiac tamponade have been met [10,11]. In this context, reports of cardiac arrhythmias have been made. As a result of myocardial inflammation and potential extensive necrosis, re-entry points in the electrical circuit may occur, leading to ventricular tachycardia and ventricular fibrillation [12,13,14]. Troponin levels have been associated with the worst outcomes, while other independent factors such as higher levels of leukocytes, D-dimer, CRP, ferritin, and IL-6 have been recognized in patients with severe COVID-19 disease. It is still unclear if myocarditis results from direct viral infection or is caused by an overactive immune reaction to the virus [15]. Additionally, increased catecholamine production in the context of Takotsubo cardiomyopathy has been revealed due to a hyper-inflammatory state and respiratory illness and a great deal of mental stress [16,17]. Finally, studies have revealed that regions of moderate carotid atherosclerosis may be especially vulnerable to thrombus formation in individuals with COVID-19 due to the unique combination of endotheliitis and COVID-19-associated coagulopathy [18,19]. 

## 3. Cytokine Storm and ARDS in COVID-19

A challenge for physicians is the optimal management of an abnormal hyper-inflammatory state with elevated pro- and inflammatory cytokines, which could drive ARDS and has been described in numerous hospitalized COVID-19 patients [7]. To the best of our knowledge, a similar disorder has been described in juvenile Still disease, revealed as a cytokine storm leading to macrophage activation syndrome (MAS), secondary haemophagocytic lymphohistiocytosis (sHLH), or cytokine release syndrome (CRS) [6,20]. These pathological entities are more commonly driven by viral infections, autoimmune disorders, malignancy (HLH and MAS), sepsis, and the administration of chimeric antigen receptor T cell therapy (CRS) [21,22]. It is characterized by sudden fever, respiratory and kidney failure, hypotensive shock, and diffuse coagulation disorders, while laboratory examinations reveal anemia, neutrophilia, thrombocytopenia, and marked lymphopenia [23,24]. Multi-organ failure presented in many cases, while four molecular cascades were responsible for its course—complement, kinin, clotting, and fibrinolysis systems. A similar phenomenon has been observed in patients with severe COVID-19 pneumonia with primary HLH as the potential underlying cause [20,25].

**Figure 1:**
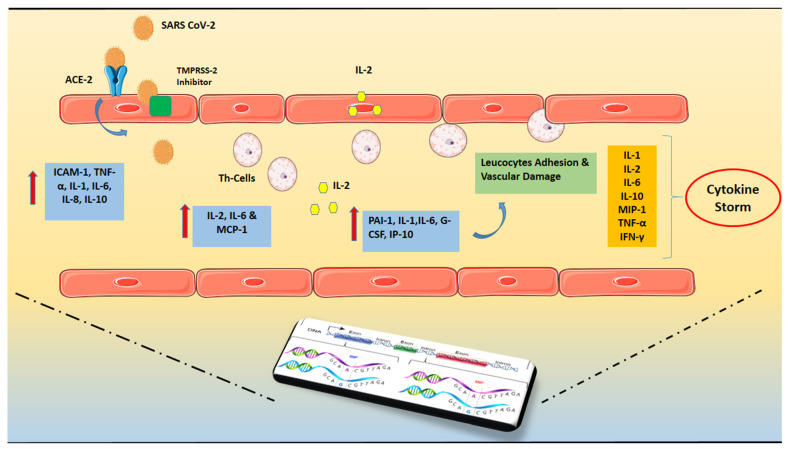
SARS-CoV-2 invasion and hyper-inflammatory state in close relation with genetic predisposition. The presence of Angiotensin-converting enzyme 2 (ACE2) and Transmembrane protease serine 2 (TMPRSS-2) that may cleave the viral spike is required for SARS-CoV-2’s cell invasion. Increased levels of pro-inflammatory cytokines, particularly the soluble interleukin 2-receptor (IL-2R) and interleukin-6 (IL-6) have been found. Soluble IL-2R (sIL-2R) is mostly released by activated T helper lymphocytes, although it may also be secreted by endothelial cells (ECs). The capillary leak is caused by the binding of IL-6 and IL-2 to their receptors. The persistent burdening of the endothelium results in increased release of inflammatory cytokines and immune system overreaction, resulting in the so-called “cytokine storm”. The above mentioned hyper-inflammatory state is in close relation with the individual genetic profile which can potentially govern the course of the disease. Abbreviations: SARS CoV-2 = Severe acute respiratory syndrome Coronavirus-2, ACE2 = Angiotensin-converting enzyme 2, TMPRSS = Transmembrane protease serine 2, IL = Interleukin, ΡAΙ-1 = Plasminogen activator inhibitor-1, TNF = Tumor Necrosis Factor, ICAM = Intercellular Adhesion Molecule 1, MCP-1 = monocyte chemoattractant protein-1, G-CSF = Granulocyte colony-stimulating factor, IP-10 = Interferon gamma-induced protein 10, MIP-1 = Macrophage inflammatory protein-1, IFN = Interferon.

The assumed underlying mechanism is the rocket of secreted inflammatory cytokine levels in the bloodstream of the patients. The critical pathogenic cytokines appear to vary according to illness, with IL-1β having an orchestrating role in Still disease, IL-18 in MAS, and IL-6 in CRS [26]. As far as the severe COVID-19 disease is concerned, the widespread hypothesis is that, in the early phase, failure of perforin, natural killer cells (NK), and CD8+ cytotoxic T-cells leads to cell lysis, initiating apoptosis of virally infected cells while interferon-γ (IFN-γ) causes excessive macrophage activation [25,27]. Multiple studies have revealed that the toll-like receptors (TLRs) and activated inflammasomes (Caspases) first release the primarily inflammatory component of the disease, IL-1β [28,29]. Parallel delayed secretion of type Ⅰ and Ⅲ IFNs, including IFN α/ß, in the early phase of infection and excessive secretion of pro-inflammatory cytokines from mononuclear macrophages is described in the later stage [30]. The cells release modest levels of antiviral factors—IFNs—as well as high amounts of pro-inflammatory cytokines—IL-1, IL-6, and TNF—and particular chemokines—C-C pattern chemokine ligand (CCL)-2, CCL-3, and CCL-5 [31,32]. Linear association of disease severity course and the type of elevated cytokines has not been well established yet. Several studies suggest that higher levels of IL-1β, IL-1RA, IL-7, IL-8, IL-10, IFN-ɣ, MCP-1, MIP-1α, G-CSF, and TNF-α have been observed in severe infection with marginal statistical significance [27,33]. Airway and alveolar epithelial cell apoptosis was induced by IFN-αβ and IFN-γ, increasing the inflammatory cell infiltration. Apoptosis of endothelial and epithelial cells affects the pulmonary microvascular and alveolar epithelial cell barriers, causing vascular leakage, alveolar edema, and, eventually, hypoxia [7,25]. The studies mentioned above emphasize that a failure in initial type-Ⅰ and Ⅲ IFN responses to SARS-CoV-2 leads to an excessive late immune response and severe form of COVID-19. The pro-inflammatory feed-forward loop of cytokines on innate immune cells results in a cytokine storm, coagulopathy, and acute respiratory distress syndrome (ARDS) [34,35]. On the other hand, in contrast with the widespread hypothesis of failure of the immune system, two other studies presented an interesting and quite different concept of cytokine storm onset, differentiating it into two stages. In the first, a short-term immune-deficient state is considered, followed by a second overactive immune condition which tries to counterbalance the agitated entropy from temporary immune target failure, driving to a cytokine storm [7,36]. As such, further molecular research on patients presented with cytokine storm caused by COVID-19 is required to clarify the phenomenon (Figure 1).

## 4. Genetic Predisposition

Many researchers support the position that cytokine storm in COVID-19 is related to individual genetic predisposition and vulnerability. This theory was strengthened by the evidence of genetic vulnerability in patients marked by primary HLH or cytokine storm in Still disease [14]. Failure of perforin and the activation of NK and cytotoxic T lymphocytes have been demonstrated as the main components of these pathologic entities.

Several studies have reported associations between human genes and COVID-19. ABO blood groups have been assessed in susceptibility to SARS-CoV-2, revealing a higher risk of infection for blood group A than non-A and a lower risk of infection for blood group O compared to non-O [37]. It is assumed that the formation of neutralizing antibodies against protein-linked N-glycans or indirect effects such as the stability of the von Willebrand factor have a partial impact on differing susceptibility [38,39,40]. Although the O blood type group seems to have an advantage concerning the susceptibility to SARS-CoV-2, there was no association between the ABO blood group and the severity of COVID-19 disease or mortality rate according to a recent meta-analysis [41]. As discussed above, invasion of SARS-CoV-2 is dependent on ACE2 and the transmembrane serine protease (TMPRSS2) [42]. Polymorphisms on the gene of *ACE2* have been associated with adverse cardiovascular and pulmonary conditions in severe COVID-19 patients due to alterations to angiotensinogen. Furthermore, the localization of the *ACE2* gene on the X chromosome may contribute to the generally higher burden observed in males as compared to in females [42]. On the other hand, a recent study illustrated that several *ACE2* variants including *K31R*, *N33I*, *H34R*, *E35K*, *E37K*, *D38V*, *Y50F*, *N51S*, *M62V*, *K68E*, *F72V*, *Y83H*, *G326E*, *G352V*, *D355N*, *Q388L*, and *D509Y* have less affinity to bind SARS-CoV-2 [43]. Although large cohort studies have not been completed yet and these variants are rare in the general population, this observation could be a cornerstone in the management of the disease [43]. In different populations, no polymorphisms or mutations associated with S binding protein have been documented [43]. Another analysis suggested that polymorphisms including *rs233574*, *rs2074192*, and *rs4646188* would change COVID-19 binding to ACE2 expressing a protective profile [44]. Of interest is the fact that three well-known polymorphisms of ACE2 (*p.(Asn720Asp)*, *p.(Lys26Arg)*, and *p.(Gly211Arg)*, as well as two rare variants *p.(Leu351Val)* and *p.(Pro389His)* have been identified and associated with a better course of the disease [45]. TMPRSS2 enzyme activity is important for coronavirus spread and pathogenesis in the infected host. The polymorphism *p.Val160Met (rs12329760)* seems to be related to increased susceptibility to SARS-CoV-2 while the oncogenic role of *TMPRSS2* may be linked to poor disease outcomes [46].

The *apolipoprotein E* (*ApoE) e4e4* homozygous genotype has been observed to enhance the risk of severe COVID-19, regardless of prior dementia, cardiovascular illness, or type 2 [47,48,49]. *ApoE e4* rules the macrophage pro-/anti-inflammatory phenotypes, and it is expressed in type II alveolar cells in the lungs where the ACE2, which SARS-CoV-2 employs for cell entrance, is abundantly co-expressed [48,49]. Association of major histocompatibility complex (MHC) class I genes (human leukocyte antigen [HLA] A, B, and C) and the susceptibility to SARS-CoV-2 have been observed. More specifically, assessing the binding affinity across HLA phenotypes and viral peptides, it is shown that harbors of HLA-B*46:01, HLA-A*11:01, -B*51:01, -C*14:02, HLA-DRB1*15:01, -DQB1*06:02, and -B*27:07 alleles are more vulnerable to SARS-CoV-2, and these mutations predispose patients to a worse disease course [50]. Loss-of-function variants of the X chromosomal *Toll-Like Receptor 7* (*TLR7*) gene have been described in individuals. The primary pathophysiologic mechanism predisposing patients to severe COVID-19 disease is the impaired type I and II IFN response and the retarded immune system reaction [50]. A wide genetic analysis of blood samples of 332 COVID-19 patients in China revealed that the most significant gene loci related to disease severity were the *Transmembrane protein 189* and *Ubiquitin Conjugating Enzyme E2 V1 (TMEM189-UBE2V1)* which play an orchestrating role in the IL-1 signaling pathway [51]. As far as the complement protein system is concerned, polymorphisms such as *C3 FF*, *C3 FS*, and *C3 SS* have been recognized as potentially deleterious for the susceptibility to SARS-CoV-2 and the course of the disease. These preliminary data should be confirmed by the large ongoing SOLID-C19 trial [52]. Finally, the *Solute Carrier Family 6 Member 20 (SLC6A20)*, *Leucine zipper transcription factor like 1 (LZTFL1)*, *C-C chemokine receptor type 9 (CCR9)*, *FYVE and Coiled-Coil Domain Autophagy Adaptor 1 (FYCO1)*, *C-X-C Motif Chemokine Receptor 6 (CXCR6)*, and *X-C Motif Chemokine Receptor 1 (XCR1)* are genes highly expressed in human lung cells, and polymorphisms have been related to the increased risk of respiratory failure and ARDS in severe COVID-19 cases [53,54] (Table 1).

## 5. Anti-Inflammatory Agents and Treatment Options

### 5.1. Inflammatory Inhibitors

#### 5.1.1. Inhibition of IL-1 Signaling

Several pro-inflammatory cytokines have been studied to find therapeutic options in severe COVID-19 cases. IL-1 is a pro-inflammatory cytokine comprised of two ligands, IL-1α and IL-1β. IL-1 promotes inflammation by recruiting immune cells and inducing secondary cytokine production, culminating in acute phase responses. Anakinra is a modified form of the human IL-1 inhibitor [55], which targets the IL-1 family, and studies showed decreased CRP and IL-6 levels, markers related to a substantial increase in mortality in severe COVID-19 illness [56,57]. In a cohort trial, patients with COVID-19 and ARDS who received Anakinra lived without the requirement for non-invasive ventilation outside of the ICU, and high-dose therapy was safe and linked with clinical improvement in 72% of patients [58]. Observational research discovered that taking Anakinra in addition to methylprednisolone decreased mortality in individuals with hyperinflammation, respiratory dysfunction, or who were on mechanical ventilation [59]. Finally, Canakinumab could be a beneficial IL-1 antagonist with research showing improvement in oxygen-support requirements and overall mortality rates [60]. Canakinumab’s effectiveness for COVID-19 needs additional investigation in randomized controlled trials.

#### 5.1.2. Inhibition of IL-6 Signaling

Infection and tissue injury provoke the secretion of IL-6, which promotes the differentiation of B and T cells, the production of acute-phase proteins, and the regulation of hepcidin levels. As such, IL-6 is a principal pro-inflammatory component that regulates the native immune response. Tocilizumab, an IL-6 receptor antagonist that inhibits signal transduction by binding soluble IL-6R and membrane-bound IL-6R and can be used in patients with bilateral pneumonia caused by SARS-CoV19, is one of the IL-6 options [61]. It was formally included in the National Health Commission of China’s COVID-19 diagnosis and treatment program and was recently approved by the Infectious Diseases Society of America. Clinical data demonstrated that most patients’ clinical course, hypoxygenemia, and computed tomography opacity alterations improved rapidly following Tocilizumab therapy [62]. The medication was associated with better overall recovery but more extended hospital stays due to unfavorable metabolic, respiratory, and infectious complications [61,62,63,64]. In a study that included 1351 patients, the use of Tocilizumab showed a significant decrease in the risk of mechanical ventilation or death. Of interest is the fact that the agent was associated with a 55% increase in survival rate in patients with mechanical ventilation needs [65]. Finally, Sarilumab, another anti-human IL-6 receptor monoclonal antibody previously used to treat rheumatoid arthritis, was found to be effective in patients with COVID-19 and hospitalization. A preliminary analysis of a trial in the United States found that Sarilumab was helpful in severely ill COVID-19 patients (those who needed mechanical breathing or high-flow oxygenation or who needed to be treated in an intensive care unit) [66].

#### 5.1.3. Inhibition of TNF-α Signaling

During the acute inflammatory state, macrophages, as well as monocytes, B cells, and other tissues, can produce TNF-α. Activation of TNF-α induces the secretion of IL-1 and IL-6. As such, it stands to reason that drugs targeting this cytokine would be of great benefit throughout the disease [67]. Although increased levels of TNF-α have been revealed in various types of cytokine storms, no significant benefit was observed considering the treatment of the phenomenon. Etanercept, Adalimumab, and Infliximab have to be administered as soon as possible from the onset of symptoms since delayed therapy may reduce the efficacy of these agents [68,69]. Although no studies have been completed, preliminary data show that it could be used to treat mild COVID-19 pneumonia to reduce IL-1 and IL-6 levels as possible endpoints [68,69].

#### 5.1.4. Inhibition of IFN-γ Signaling

IFN-γ has been recognized as a critical pro-inflammatory component in various cytokine storms such as primary HLH [70]. Its ability to stimulate the inflammatory response has made it an exciting research topic, particularly during the COVID-19 era. Since its FDA approval in 2018, Emapalumab, an anti-IFN-monoclonal antibody, has been used to treat primary HLH. Trials showed a significant reduction of C-X-C motif chemokine ligand 9 (CXCL9) levels in the bloodstream with clinical improvement. Trials on COVID-19 patients using Emapalumab are ongoing with hopes that it could be a valid treatment option [71].

#### 5.1.5. Inhibition of JAK Pathway

The Janus kinase-signal transducer and activator of transcription (JAK/STAT) pathway is commonly involved in various cytokine activation processes [72]. JAK is an intracellular tyrosine kinase that mediates cytokines, hormones, and growth factor signals. The use of JAK inhibition in COVID-19 patients will potentially reduce the inflammatory burden, inhibiting the secretion of common inflammatory cytokines (IL-1, IL-6, TNF-a, etc.), and impeding the entry and proliferation of SARS-CoV-2 [73]. The latter can be explained by the ability of JAK inhibitors to reduce the action of the AP2-associated protein kinase-1 regulator of SARS-CoV-2 invasion in alveolar epithelial cells. Studies on COVID-19 patients have illustrated rapid clinical improvement with Ruxolitinib or Fedratinib treatment while Baricitinib reduced the rate of intensive care unit admission and fatality and increased discharge rates [72,73,74].

#### 5.1.6. Inhibition of Granulocyte–Macrophage Colony-Stimulating Factor Signaling

Alveolar epithelial cells secrete GM–CSF, which can act as a growth factor and pro-inflammatory cytokine. GM–CSF regulates the native immune response in the lungs driving pulmonary host defense function against pathogens [75]. In severe COVID-19 patients, particularly during hyper-inflammation state and cytokine storm, GM–CSF levels have been found to be unexpectedly high. As such, and considering the upregulation of inflammatory cytokines and chemokines levels via GM–CSF’s action, downregulation of this ligand would be potentially beneficial [76,77]. Multiple trials are trying to assess the efficiency of monoclonal antibodies neutralizing GM–CSF as possible treatments for preventing and curing ARDS, such as Gimsilumab, Lenzilumab, Namilumab, and TJM2 targeting to decrease cytokine levels and mortality rates [76,77].

### 5.2. Other Non-Specific Anti-Inflammatory Agents

#### 5.2.1. Corticosteroids

Corticosteroids are effective cytokine inhibitors, primarily suppressing the nuclear factor kappa-light-chain-enhancer of the activated B cell (NF-B) transcription factor [3]. Furthermore, corticosteroids seem to have anti-fibrotic properties with good patient tolerance [78]. Corticosteroids inhibit the production of pro-inflammatory cytokines, avoiding a protracted cytokine response and hastening recovery from pneumonia. Since it is well known that corticosteroids downregulate the native immune response with wide spectrum immunosuppression, studies recommend their use on a low dose (≤0.5–1 mg/kg/day of methylprednisolone or equivalent) and short duration (≤7 days) for patients with severe cases of COVID-19 or ARDS. It has been observed that dexamethasone treatment helps SpO2 levels approach more than 90% in each case. Furthermore, it reduces hospitalization, intubation, and the number of patients who do not require mechanical ventilation [3,79]. The RECOVERY trial showed a significant reduction of mortality risk in the group of patients on mechanical ventilation (by one-third) receiving oxygen (by one-fifth) when applied to the administration of low-dose dexamethasone (6 mg once daily, orally or intravenously) for ten days. The death rates during hospitalization and one month after discharge were reduced [3,79,80]. Corticosteroids were related to decreased all-cause mortality at 28 days in a recent meta-analysis of 1703 critically ill patients with COVID-19. There was no evidence of an increased risk of side effects, and survival rates were comparable in the dexamethasone and hydrocortisone groups [3]. The tone of physicians’ awareness is the higher mineralocorticoid activity of methylprednisolone, which indicates a strict control of fluid and sodium administration. A larger dose of corticosteroids did not provide more advantages than a smaller dose [3].

#### 5.2.2. Statins

COVID-19 inflammatory cytokines and chemokines share many similarities with those seen in the early and late phases of atherosclerotic plaque development [81,82]. Statins’ anti-inflammatory properties are supported by their action on the mevalonate pathway, which influences endothelial function, inflammation, and coagulation [83]. Furthermore, statins inhibit the overexpression or underexpression of the myeloid differentiation primary response 88 (*MyD88*) gene by keeping its level in the bloodstream relatively stable. Alterations in the expression of *MyD88* have already been linked with higher mortality rates and vulnerability in COVID-19 disease [84,85]. Finally, statins downregulate the secretion of pro-inflammatory cytokines such as CRP and IL-6 in animal models, while they have become the gold standard therapy for cardiovascular risk reduction and prevention [86].

#### 5.2.3. Colchicine-Macrolides

Colchicine’s anti-inflammatory properties are based on two mechanisms: (a) direct inhibition of (NOD)-like receptor protein 3 (NLRP3) inflammasome and, consequently, of IL-1β production, and (b) inhibition of the polymerization of microtubes [87]. Although it was considered a novel therapeutic approach for COVID-19 patients, no established benefit has yet been met [88,89]. On the contrary, dose-dependent inhibition of surfactant by affecting alveolars types II pneumocytes has been observed, consequently burdening ARDS patients with potential multiorgan failure and disseminated intravascular coagulation [90,91].

**Figure 2:**
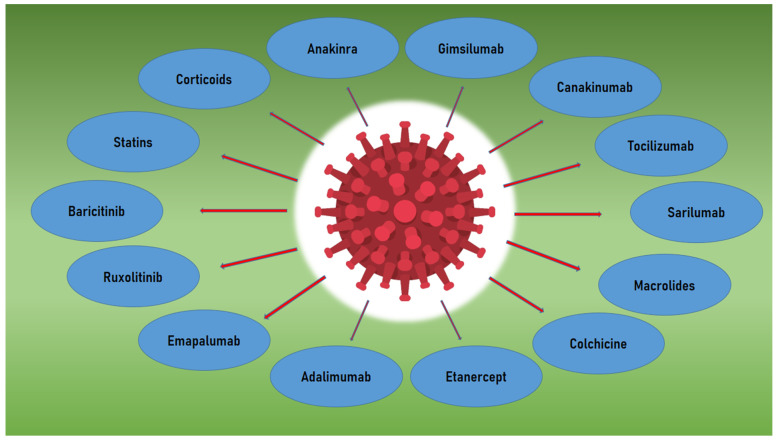
Graphical illustration of the treatment options in COVID-19 disease and the agents under assessment.

Macrolides such as azithromycin and clarithromycin are agents with high patient tolerance. Their anti-inflammatory and immunomodulatory effects are used widely in cases of viral pneumonia as adjunctive therapy to prevent bacterial infection [92,93]. In the COVID-19 era, azithromycin is used daily for its anti-inflammatory and bacterial preventive properties, but the exact benefit is not fully understood [92,93]. The use of azithromycin in COVID-19 patients is not well established and is still debated, while adverse effects such as prolongation of the QT interval could potentially increase the risk of sudden cardiac death in non-monitored patients [94] (Table 2, Figure 2).

## 6. Conclusions

SARS-CoV-2 and the resulting COVID-19 have led to a global pandemic with catastrophic outcomes due to the induced hyperinflammatory state and systemic endothelial activation, which result in pulmonary and cardiovascular complications. Genetic predisposition, as in other inflammatory diseases, might be responsible for alterations in the clinical course of COVID-19 patients through polymorphisms in crucial genes such as ACE2 and MHC class I. Components of the immune response to the virus appear to be primarily related to disease severity, whereas genes related to the binding of the ACE2 cell surface—the entry point for SARS-CoV-2—during the early stages of infection appear to be largely responsible for the varying susceptibility to SARS-CoV-2. Inflammatory inhibitors are at the forefront of pharmacological management in COVID-19, although their potential has not been fully elucidated till now. The above mentioned would have a potentially large impact on targeted medicines and, more critically, vaccine development. Moving forward, we must acknowledge that each decade of the twenty-first century has seen a new significant coronavirus epidemic: SARS in the 2000s, MERS in the 2010s, and now COVID-19. As such, evidence-based risk assessment might result in individualized preventive methods and treatment approaches while further SARS-CoV-2 molecular research is needed to develop more sensitive genetic-based detection and treatment methods to overcome the pandemic.

## Figures and Tables

**Table 1:** Genetic polymorphisms under assessment in COVID-19 disease.

Gene	Polymorphism	Result
*ABO*	*rs657152*	Higher risk of infection for blood group A vs. non-A and lower risk of infection for blood group O vs. non-O [37].
*HLA*	HLA-B*46:01, HLA-A*11:01, -B*51:01, -C*14:02, HLA-DRB1*15:01, -DQB1*06:02, and -B*27:07	Vulnerable to disease for HLA-B*46:01 and cross-protective T cell-based immunity for HLA-B*15:03 [50].
*TMPRSS2*	*p.Val160Met (rs12329760)*	Increased susceptibility to SARS-CoV-2 [46].
*ACE2*	*K31R, N33I, H34R, E35K, E37K, D38V, Y50F, N51S, M62V, K68E, F72V, Y83H, G326E, G352V, D355N, Q388L, and D509Y* *rs233574* *rs2074192* *rs4646188* *(p.(Asn720Asp)* *p.(Lys26Arg) p.(Gly211Arg)* *p.(Leu351Val)* *p.(Pro389His)*	Better cardiovascular and pulmonary course of the disease, less susceptibility to SARS-CoV-2 [42,43,44,45].
*ApoE*	*rs429358-C-C (e4e4)*	Severe course of the disease [48,49].
*SLC6A20, LZTFL1, CCR9, FYCO1, CXCR6, XCR1*	*rs11385942-GA*	Severe course of the disease and potentially higher odds for ARDS [53,54].
*Of Complement proteins*	*C3 FF, C3 FS, C3 SS*	Severe course of the disease, Increased susceptibility to SARS-CoV-2 [52].
*TMEM189- UBE2V1*	*rs6020298-A*	Severe course of the disease [51].
*TLR7*	*g.12905756_12905759del and g.12906010G > T*	Severe course of the disease [50].

Abbreviations: ApoE = Apolipoprotein E, ACE2 = Angiotensin-converting enzyme 2, TMPRSS2 = Transmembrane Serine Protease 2, HLA = Human Leukocyte Antigen, ABO = ABO blood system, TLR7 = Toll-like receptor 7, TMEM189-UBE2V1 = Transmembrane protein 189 and Ubiquitin Conjugating Enzyme E2 V1, SLC6A20 = Solute Carrier Family 6 Member 20, LZTFL1 = Leucine zipper transcription factor like 1, CCR9 = C-C chemokine receptor type 9, FYCO1 = FYVE And Coiled-Coil Domain Autophagy Adaptor 1, CXCR6 = C-X-C Motif Chemokine Receptor 6, XCR1 = X-C Motif Chemokine Receptor 1.

**Table 2:** Anti-inflammatory therapeutic options for COVID-19 disease.

Agent	Dose-Route of Administration	Action	Specific Populations	Adverse Events	Contraindications
Anakinra	IV: 100 mg every 6 h (total daily dose: 400 mg) for 15 days; 200 mg every 8 h for 7 days; 300 mg od for 4 days, followed by 100 mg od SC: 100 mg od for 10 or 28 days. Alternative regimen: 100 mg every 12 h on days 1–3, then 100 mg od from days 4–10	IL-1 receptor antagonist	Higher rates of infections in the elderly population In patients with CrCl < 30 and ESRD, use extended dosing intervals (every other day)	Injection site reactions, upper respiratory tract infections, headache, nausea, diarrhea, sinusitis, flu-like symptoms, abdominal pain	Hypersensitivity to *Escherichia* *coli*-derived proteins
Canakinumab	Undefined/IV/It is administered every eight weeks as a single dose via subcutaneous injection	IL-1 receptor antagonist	Canakinumab has not been studied in patients with hepatic impairment	Respiratory tract infections (including pneumonia, bronchitis, influenza, viral infection, sinusitis, rhinitis, pharyngitis, tonsillitis, nasopharyngitis, upper respiratory tract infection) Ear infection Cellulitis Gastroenteritis Urinary tract infection	Hypersensitivity
Tocilizumab	IV: 4–8 mg/kg (maximum single dose: 800 mg), may repeat after 12 h	IL-6 receptor antagonist	Safety during pregnancy and lactation is unknown	Injection site reactions, upper respiratory tract infections (including tuberculosis), nasopharyngitis, headache, hypertension, increased ALT, hematological effects	Hypersensitivity
Sarilumab	Undefined/IV	IL-6 receptor antagonist	Safety during pregnancy and lactation is unknown	Neutropenia, increased ALT, injection site erythema, upper respiratory infections, urinary tract infections	Hypersensitivity
Etanercept	Undefined/IV	TNF-a inhibitor	High awareness in pediatric population	Pain, swelling, itching, reddening, and bleeding at the puncture site, infections (such as upper respiratory infections, bronchitis, bladder infections, and skin infections), headache, allergic reactions, development of autoantibodies, itching, and fever	Hypersensitivity, Sepsis, Not be initiated in patients with active infections, including chronic or localized infections
Adalimumab	Undefined/IV	TNF-a inhibitor	Use with caution in patients with heart failure or decreased left ventricular function; may cause myocardial toxicity or exacerbate underlying myocardial dysfunction Use caution in elderly infection risk patients; may increase	Upper respiratory tract infections, sinusitis, increased macrophage- dependent infection, tuberculosis, opportunistic infections, injection site reactions, increased creatine phosphokinase, headache, rash	None
Emapalumab	Undefined/IV	TNF-a inhibitor	None	Infections, hypertension, infusion-related reactions, and pyrexia.	None
Ruxolitinib	Various regimens under investigation: PO: 5 mg bid for 14 days; 10 mg bid; 2 × 10 mg bid dose at day 1 and can be increased up to 2 × 15 mg bid from day 2 to day 28; 5 mg bid from day 1 to day 3, then 10 mg bid from day 4 to day 10; 10 mg bid, for 14 days followed by 5 mg bid for 2 days and 5 mg od for 1 day	JAK1/ JAK2 inhibitor	Use in pregnant and lactating women is not recommended May require starting dose reduction in hepatic and renal impairment	Thrombocytopenia, neutropenia, anemia, infections, edema, headache, dizziness	None
Baricitinib	PO: 2 or 4 mg od for 14 days	JAK1/ JAK2 inhibitor	Avoid use in patients with severe hepatic impairment, and in patients with moderate or severe renal impairment	Upper respiratory tract infections, nausea, herpes simplex, herpes zoster	None
Gimsilumab	IV: High dose on day 1 and low dose on day 8, specifics not described	Anti-GM–CSF	None	None	None
Dexamethasone	IV or PO: RECOVERY trial: 6 mg daily for 10 days; DEXACOVID19 trial: 20 mg od from day 1 to day 5, followed by 10 mg od from day 6 to day 10	Anti-inflammatory and anti-fibrotic effects	Use with caution in the elderly with the smallest possible effective dose for the shortest duration	Sodium and water retention (less than methylprednisolone), hypertension, hyperglycemia, osteoporosis, cardiac hypertrophy, edema, hypokalemia, bruising, diaphoresis, urticaria, allergic rash, euphoria, psychosis, infections, myasthenia gravis	Hypersensitivity to corticosteroids or any component of the formulation, systemic fungal infection
Methylprednisolone	IV: 0.5–1 mg/kg daily or 1–2 mg/kg daily (of methylprednisolone or equivalent) have been proposed Higher doses (cytokine storm): 60–125 mg (methylprednisolone) every 6 h for up to 3 days	Anti-inflammatory and anti-fibrotic effects	Use with caution in the elderly with the smallest possible effective dose for the shortest duration	Sodium and water retention, hypertension, hyperglycemia, osteoporosis, cardiac hypertrophy, edema, hypokalemia, bruising, diaphoresis, urticaria, allergic rash, euphoria, psychosis, infections, myasthenia gravis	Hypersensitivity to corticosteroids or any component of the formulation, systemic fungal infection
Statins	PO: Simvastatin 40 mg od for 14 days, simvastatin 80 mg od, atorvastatin 40 mg od	Anti-inflammatory and pleiotropic effects	Use with caution in elderly patients; may be at higher risk for myopathy	Hepatotoxicity, myopathies, GI effects, rhabdomyolysis, increased risk of diabetes	Hypersensitivity to Statin, active liver disease; unexplained persistent elevations of serum transaminases; pregnancy, breastfeeding
Colchicine	PO: 0.5 mg bid for 3 days, then 0.5 mg od for 27 days	Anti-inflammatory and immunomodulatory effects	Dose adjustment is required in patients with renal or hepatic function	GI symptoms (diarrhea, nausea, vomiting, abdominal pain), neuromuscular toxicity, hematological effects, elevated AST and ALT	Renal or hepatic impairment in conjunction with drugs that inhibit both CYP3A4 and P-gp (e.g., clarithromycin)
Azithromycin	PO: 500 mg on day 1, then 250 mg od on days 2–5	Anti-inflammatory and bacteriostatic effects	Torsades de pointes Arrhythmias in elderly patients	QTc prolongation and ventricular arrhythmias, diarrhea, nausea, abdominal pain, vomiting	Hypersensitivity to azithromycin or other macrolides, history of cholestatic jaundice/ hepatic dysfunction associated with prior azithromycin use
Clarithromycin	PO: 250 mg twice daily or 500 mg twice daily in severe cases	Anti-inflammatory and bacteriostatic effects	Parallel administrarion with astemizole, cisapride, pimozide, and terfenadine may result in QT prolongation and cardiac arrhythmias, including ventricular tachycardia, ventricular fibrillation, and Torsades de pointes	Abdominal pain, diarrhoea, nausea, vomiting and taste perversion	Hypersensitivity to Clarithromycin or other macrolides, Clarithromycin should not be used in patients who suffer from severe hepatic failure in combination with renal impairment

## Data Availability

Not applicable.
